# Simulation of English Word Order Sorting Based on Semionline Model and Artificial Intelligence

**DOI:** 10.1155/2022/5999853

**Published:** 2022-06-28

**Authors:** Zhange Meng, Nan Li

**Affiliations:** Xingtai University, Xingtai, Hebei, China

## Abstract

To improve the word order ranking effect of English language retrieval, based on machine learning algorithms, this paper combines a semionline model to construct an artificial intelligence ranking model for English word order based on a semionline model and establishes a semisupervised ELM regression model. Moreover, this paper derives the mathematical model of semisupervised ELM in detail and uses FCM clustering to screen credible samples, ELM collaborative training to mark each other's samples, and the marked samples to calculate the output weights of semisupervised ELM regression. In addition, based on continuous learning of OSELMR, this paper uses confidence evaluation to screen out credible unlabeled samples, OSELM collaborative training to mark the credible samples with each other, and credible unlabeled samples to calculate the output weight of SSOSELMR. Finally, this paper designs a control experiment to analyze the model algorithm, compares and counts the parameters, and draws a statistical graph. The research results show that the model constructed in this paper is effective.

## 1. Introduction

English has become the universal language in the world, and there will inevitably be problems with English ordering when obtaining information, which also directly affects the terminal experience of nonnative English speakers. To improve the effect of English word order, artificial intelligence models need to be used for auxiliary improvement [1]. Information retrieval research usually covers two issues that need to be solved urgently. One is the understanding of user queries, that is, how to fully understand the user's information needs as much as possible based on the user's submitted queries that only contain a few keywords, and provide documents or web pages that meet their needs [2]. The second is the construction of the retrieval model, that is, how to measure the degree of relevance between the user's query and the web page or document to be retrieved and give a sorted list of results according to the difference in relevance, to meet the user's information needs [3]. To solve the previously mentioned two problems, information retrieval research is usually carried out from two aspects. On the one hand, research focuses on fully understanding user queries, and on the other hand, research focuses on the reasonable construction of retrieval models. The previously mentioned two researches are complementary, and a full understanding of user queries can enable the retrieval system to more accurately locate user information needs. On this basis, a reasonably constructed retrieval model can mine web pages or documents related to user needs to meet information needs and improve retrieval accuracy and user experience [4].

In query expansion or query reduction, the direct addition or deletion of some terms is likely to cause part of the user's information needs to be missing or offset. To avoid this kind of situation, a common practice is to weigh the query terms. That is, based on the reconstruction of the query, the terms that are more closely related to the user's information needs are given a higher weight, and the terms that are more ambiguous with the user's information needs are given a lower weight, so as to fully cover the user's information needs while taking into account the completeness of the query, and understand the user's query more accurately [5]. In terms of the retrieval model, the traditional retrieval model aims to construct effective query representation and document representation. It evaluates the relevance between the document and the query by calculating the similarity between the two and then gives a sorted list of documents as the output result of the retrieval system according to the relevance of the document from high to low. Such methods include the vector space model, BM25 model, and query likelihood language model [6]. Next, this article takes the vector space model as an example. The model represents the query and document as vectors of dictionary dimensions. Among them, the dictionary dimension refers to the total number of all terms included in the retrieval data set, and the value of each dimension of the vector is the weight of the term. The weight calculation of this model can adopt such methods as word frequency inverse document frequency [7]. Furthermore, the model calculates the similarity between the query and the document based on the query representation and the document representation and uses this as a basis to evaluate the relevance of the query and different documents to obtain the document ranking list [8]. In recent years, learning to rank (LTR) has been proposed and widely used to construct more effective retrieval models. Different from the traditional retrieval model, the ranking learning model uses a supervised machine learning method as the core algorithm. Moreover, it takes traditional retrieval model scores as document features, takes document ranking as model optimization goal, defines a loss function based on ranking, and obtains the final retrieval model through supervised training [9].

## 2. Related Work

To solve the inconsistency between the original query and the expanded term, the literature proposed to update the query language model with pseudorelevant feedback documents [10]. Moreover, it verified the effectiveness of the model within the framework of two retrieval methods: probabilistic model feedback and KL distance minimization. The literature proposed to predict query performance by calculating the relative entropy of the query language model and the collective language model and used the clarity index to predict the degree of ambiguity of query expression [11]. The literature regarded query expansion as an optimization problem integrating multiple information sources and multiple goals and gave detailed theoretical derivation and comprehensive empirical evaluation [12]. Moreover, it reduced the empirical risk of query expansion from the robustness limitation, which lays an important theoretical foundation for subsequent research on query expansion. Aiming at the problem of parameter adjustment in pseudocorrelation feedback, the literature proposed a pseudocorrelation feedback method based on statistical language models [13]. This method integrates the original query and feedback documents through a single probability mixed model and uses language model parameters as regularization items, thereby effectively avoiding manual selection of parameters, improving the robustness of retrieval results and the generalization ability of the method. The literature proposed to use word vectors to expand the query language model. This model uses word vectors as nonquery word selection and weight evaluation and applies this method to the pseudorelevance feedback process [14]. The experimental results show that this method can select more semantically related expansion words to improve retrieval performance. The literature proposed a probabilistic ranking function based on Bayesian decision theory, which is used to fuse document language models and query language models, and used Markov random chain prediction query language models based on document collections [15].

## 3. Extreme Learning Machine

**Figure 1:**
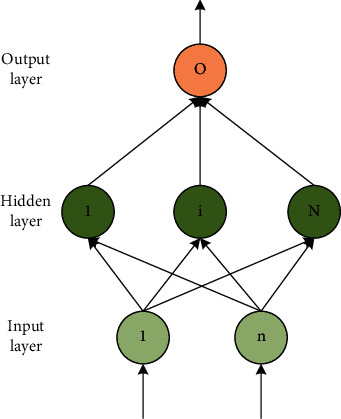
Single hidden layer feedforward neural network.

The artificial neural network is composed of the sensations of analog neurons connected to each other. According to the connection mode of the perceptron, the neural network can be divided into a feedforward neural network and a feedback neural network. The feedforward neural network has a three-layer network structure: input layer, hidden layer, and output layer. Among them, the hidden layer can have several layers. The learning process of the neural network is that the input layer inputs the observation value, the hidden layer trains the observation value to obtain the estimated value of the parameter, and the output layer outputs the target value of the sample. The single hidden layer feedforward neural network is a feedforward neural network with only one hidden layer. The network structure of SLFN is shown in Figure 1. For the generality of the analysis, the figure describes the situation where the output layer is a target output.

As shown in Figure 1, in the standard SLFN, $\tilde{N}$ is the number of hidden layer nodes, *G* is the activation function, and *X* is the input sample of the input layer, which has *n* attributes of gates. *w*_*i*_ and *b*_*i*_ are the front parameters of the hidden layer, and the subscript *i* is the *i*-th node in the hidden layer. For increasing the node SLFN, *w*_*i*_ is the weight connecting the input layer node and the *i*-th node in the hidden layer, and *b*_*i*_ is the threshold value of the *i*-th node in the hidden layer. For the radial base node SLFN, *w*_*i*_ is the center of the radial base node, and *b*_*i*_ is the influence factor of the radial base node.

The output of the *i*-th node in the hidden layer of the added node SLFN is

(1) $$\begin{array}{c} G\left(w_{i},b_{i},x\right)=g\left(w_{i}\cdot x+b_{i}\right). \end{array}$$

The output of the *i*-th node of the hidden layer of the radial basis node SLFN is

(2) $$\begin{array}{c} G\left(w_{i},b_{i},x\right)=g\left(b_{i}\left\|x-w_{i}\right\|\right). \end{array}$$

Furthermore, by weighting the output of $\tilde{N}$ nodes in the hidden layer, the output of the SLFN network is obtained:

(3) $$\begin{array}{c} o=f_{\tilde{N}}\left(x\right)\\ =\overset{\tilde{N}}{\underset{i=1}{\sum }}\beta_{i}G\left(w_{i},b_{i},x\right). \end{array}$$

Among them, *β*_*i*_ is the weight connecting the *i*-th hidden layer node and the output layer node, and *w*_*i*_ · *x* represents the inner product of *w*_*i*_ and *x*.

The SLFN learning process is to input the observation value of the sample into the input layer, train the attributes of the labeled sample and determine the specific values of the parameters *w*_*i*_, *b*_*i*_, and *β*_*i*_ for the target output, and use the trained parameter values to calculate the label value of the unknown label sample and the output layer to output the label value of the sample.

For *N* mutually independent and different samples (*x*_*i*_, *t*_*i*_),

(4) $$\begin{array}{c} x_{i}={\left[x_{i1},x_{i2},\ldots ,x_{in}\right]}^{T}\in R^{n},\\ t_{i}={\left[t_{i1},t_{i2},\ldots ,t_{im}\right]}^{T}\in R^{m}. \end{array}$$

The mathematical model of the standard SLFN with the number of hidden nodes being $\tilde{N}$ and the activation function being *g*(*x*) is

(5) $$\begin{array}{c} \overset{N}{\underset{j=1}{\sum }}\overset{\tilde{N}}{\underset{i=1}{\sum }}\beta_{i}g\left(x_{j}\right)=\overset{N}{\underset{j=1}{\sum }}\overset{\tilde{N}}{\underset{i=1}{\sum }}\beta_{i}g\left(w_{i}\cdot x_{j}+b_{i}\right). \end{array}$$

Among them,

(6) $$\begin{array}{c} w_{i}={\left[w_{i1},w_{i2},\ldots ,w_{in}\right]}^{T},\\ \beta_{i}={\left[\beta_{i1},\beta_{i2},\ldots ,\beta_{im}\right]}^{T}. \end{array}$$

The standard SLFN with the number of hidden layer nodes $\tilde{N}$ and the activation number *g*(*x*) can approximate *N* different samples with zero error; that is, the error between the instantaneous standard output of the standard SLFN and the real is zero:

(7) $$\begin{array}{c} \overset{\tilde{N}}{\underset{j=1}{\sum }}\left\|o_{j}-t_{j}\right\|=0. \end{array}$$

In other words, there are parameters *β*_*i*_, *w*_*i*_, and *b*_*i*_ that make the following true:

(8) $$\begin{array}{c} \overset{N}{\underset{j=1}{\sum }}\overset{\tilde{N}}{\underset{i=1}{\sum }}\beta_{i}g\left(w_{i}\cdot x_{j}+b_{i}\right)=t_{j}. \end{array}$$

The mathematical model of the standard SLFN:

(9) $$\begin{array}{c} \overset{N}{\underset{j=1}{\sum }}\overset{\tilde{N}}{\underset{i=1}{\sum }}\beta_{i}g\left(w_{i}\cdot x_{j}+b_{i}\right)=t_{j}. \end{array}$$

It is abbreviated as follows:

(10) $$\begin{array}{c} H\beta =T. \end{array}$$

Among them,

(11) $$\begin{array}{c} H\left(w_{1},\ldots ,w_{\tilde{N}},b_{1},\ldots ,b_{\tilde{N}},x_{1},\ldots ,x_{N}\right)={\left[\begin{array}{ccc} g\left(w_{1}\cdot x_{1}+b_{1}\right) & \cdots & g\left(w_{\tilde{N}}\cdot x_{1}+b_{\tilde{N}}\right)\\ \vdots & \cdots & \vdots \\ g\left(w_{1}\cdot x_{N}+b_{1}\right) & \cdots & g\left(w_{\tilde{N}}\cdot x_{N}+b_{\tilde{N}}\right) \end{array}\right]}_{N\times \tilde{N}},\\ T={\left[\begin{array}{c} t_{1}^{T}\\ \vdots \\ t_{N}^{T} \end{array}\right]}_{N\times m}. \end{array}$$

*H* is called the hidden layer output matrix of SLFN, the column of *H* is the output value of each hidden layer node corresponding to the input *x*_1_, *x*_2_,…, *x*_*N*_, and the row of *H* is the output value of the hidden layer node $1,\ldots ,\tilde{N}$ of each sample.

SLFN has the following theorems.

**Theorem 1 .**

The activation function *g*(*x*) of the standard SLFN with the hidden node $\tilde{N}$ is infinitely differentiable in any interval. The input samples are N mutually independent and different samples (*x*_*i*_, *t*_*i*_), where *x*_*i*_ ∈ *R*^*n*^, *t*_*i*_ ∈ *R*^*m*^. When random numbers on any interval of *R*^*n*^ and *R* generated by any continuous probability distribution function are assigned to *w*_*i*_*n* and *b*_*i*_, there must be a hidden layer output matrix *H* of the SLFN network that is invertible.

(12) $$\begin{array}{c} \left\|H\beta -T\right\|=0. \end{array}$$

**Theorem 2 .**

When there is an infinitely small positive number *ε* < 0, for an infinitely differentiable standard SLFN in any interval, the hidden layer node is *n* and the activation function is *g*(*x*), and N mutually independent and different samples (*x*_*i*_, *t*_*i*_) are input, where *x*_*i*_ ∈ *R*^*n*^, *t*_*i*_ ∈ *R*^*m*^, and $\tilde{N}\leq N$. When random numbers on any interval of *R*^*n*^ and *R* generated for any continuous probability distribution function are assigned to *w*_*i*_ and *b*_*i*_, there must be

(13) $$\begin{array}{c} \left\|H_{N\times \tilde{N}}\beta_{\tilde{N}\times N}-T_{N\times M}\right\|\leq \varepsilon . \end{array}$$

When the activation function of SLFN is infinitely differentiable, for a dataset containing N different samples, the number of hidden layer nodes of SLFN needs to be much smaller than the number of samples, that is, $\tilde{N}\leq N$. In addition, activation functions that are infinitely differentiable in any interval include sigmoidal, sine, and cosine.

The cost function *E* of SLFN is

(14) $$\begin{array}{c} E=\overset{N}{\underset{j=1}{\sum }}{\left(\overset{\tilde{N}}{\underset{i=1}{\sum }}\beta_{i}g\left(w_{i}\cdot x_{j}+b_{i}\right)-t_{j}\right)}^{2}. \end{array}$$

Traditionally, the process of using a dataset to train the SLFN is to specify the values of the parameters *β*_*i*_, *w*_*i*_, and *b*_*i*_, and find the estimated values of ${\hat{\beta }}_{i}{\hat{w}}_{i}{\hat{b}}_{i}$ that satisfy the following:

(15) $$\begin{array}{c} {\hat{w}}_{i},{\hat{b}}_{i},\hat{\beta }=\underset{w_{i},b_{i},\beta }{\min}\left\|H\left(w_{1},\ldots ,w_{\tilde{N}},b_{1},\ldots ,b_{\tilde{N}}\right)\beta -T\right\|. \end{array}$$

When *H* is unknown, the gradient descent algorithm is usually used to find the minimum value of ‖*Hβ* − *T*‖. In the step of using the gradient descent algorithm, (*w*_*i*_, *β*_*i*_) and *b*_*i*_ constitute a vector *W*, as shown in the following formula, and the parameter values are adjusted iteratively.

(16) $$\begin{array}{c} W_{k+1}=W_{k}-\eta \frac{\partial E\left(W\right)}{\partial W}. \end{array}$$

Among them, *η* is the learning rate.

The BP algorithm, one of the typical algorithms of feedforward neural networks, calculates the gradient by returning the output value to the input layer. However, there are some problems in the BP algorithm.

1. When the learning rate is too small, the algorithm converges slowly, and the learning rate is too large, and the algorithm is unstable or even divergent.
2. BP algorithm has a local minimum solution, and learning will stop at the local minimum solution. When the local minimum solution is far from the global minimum solution, the result is not ideal.
3. The network may be overtrained and get worse generalization ability. It is necessary to add verification and appropriate stopping conditions to the cost function.
4. In many applications, the process of gradient descent takes a long time.

Traditional SLFN needs to adjust the input weight and hidden layer threshold. According to Theorems 1 and 2, when the activation function is infinitely differentiable, the parameters *w*_*i*_ and *b*_*i*_ can be randomly assigned. Compared with traditional SLFN, all parameters need to be adjusted. In this case, there is no need to iteratively adjust the values of *w*_*i*_ and *b*_*i*_. In the initial stage of learning, random values are assigned to the parameters *w*_*i*_ and *b*_*i*_, and the output matrix H of the hidden layer remains unchanged. In the subsequent learning process, the values of parameters *w*_*i*_ and *b*_*i*_ are fixed, and the process of training SLFN is to find the least square solution $\hat{\beta }$ of the linear system *Hβ*=*T*.

(17) $$\begin{array}{c} \left\|H\left(w_{1},\ldots ,w_{\tilde{N}},b_{1},\ldots ,b_{\tilde{N}}\right)\beta -T\right\|=\underset{\beta }{\min}\left\|H\left(w_{1},\ldots ,w_{\tilde{N}},b_{1},\ldots ,b_{\tilde{N}}\right)\beta -T\right\|. \end{array}$$

When the number of input samples is equal to the number of hidden layer nodes $\tilde{N}=N$, the values of input weight *w*_*i*_ and hidden layer threshold *b*_*i*_ are randomly selected, and the matrix *H* is square and invertible, and SLFN can approximate the training samples with zero error.

However, in most cases, the number of hidden layer nodes is much smaller than the number of training samples $\tilde{N}\leq N$. Currently, *H* is not a square matrix, and there are no *β*_*i*_, *w*_*i*_, and *b*_*i*_ to make

(18) $$\begin{array}{c} H\beta =T. \end{array}$$

Currently, according to the nature of the *Hβ*=*T* solution of the linear system, the least square solution with the smallest norm is

(19) $$\begin{array}{c} \hat{\beta }=H^{+}T={\left(H^{T}H\right)}^{-1}H^{T}T, \end{array}$$

where *H*^+^ is the generalized inverse of *H*.

The following important properties of SLFN can be obtained from the previously mentioned description.

(1) The least square solution makes SLFN have the smallest training error. When $\hat{\beta }=H^{+}T$ is the least square solution of the linear system *Hβ*=*T*, the training error is the smallest as follows:
  (20) $$\begin{array}{c} \left\|H\hat{\beta }-T\right\|=\left\|HH^{+}T-T\right\|\\ =\underset{\beta }{\min}\left\|H\beta -T\right\|. \end{array}$$
(2) SLFN has an output weight with the smallest norm. $\hat{\beta }=H^{+}T$ is the least square solution with the smallest norm among all the solutions of the linear system *Hβ*=*T* as follows:
  (21) $$\begin{array}{c} \left\|\hat{\beta }\right\|=\left\|H^{+}T\right\|\leq \left\|\beta \right\|,\\ \forall \beta =\left\{\beta :\left\|H\beta -T\right\|\leq \left\|Hz-T\right\|,\forall z\in R^{\tilde{N}\times N}\right\}. \end{array}$$
(3) The least squares solution with the smallest norm of SLFN is unique.

In view of the previously mentioned analysis, an extreme learning machine (ELM) is proposed. The ELM learning process is as follows.

The training set is

(22) $$\begin{array}{c} N=\left\{\left(x_{j},t_{j}\right)|x_{j}\in R^{N},t_{j}\in R^{m},\;j=1,\ldots ,N\right\}. \end{array}$$

The activation function is *g*(*x*), and the number of hidden layer nodes is $\tilde{N}$.

(1) The input weight *w*_*i*_ and the hidden layer threshold *b*_*i*_ are randomly generated, where $i=1,\ldots ,\tilde{N}$.
(2) The hidden layer output matrix *H* is calculated.
  (23) $$\begin{array}{c} H=\overset{\tilde{N}}{\underset{i=1}{\sum }}g\left(w_{i}w_{j}+b_{i}\right). \end{array}$$
(3) The output weight $\hat{\beta }=H^{+}T$ is calculated, where *T*=[*t*_1_,…,*t*_*N*_]^*T*^.

According to the different connection modes of hidden layer nodes in ELM, ELM can be divided into increased-node-ELM and RBF-node-ELM. The activation function of the increased node ELM is an arbitrary bounded nonconstant piecewise online function, and the activation function of the RBF node ELM is an arbitrary integrable piecewise function.

Therefore, we set the number of hidden layer nodes of the ELM to $\tilde{N}$, use the data set of *N* samples to train the ELM to obtain the output weight, and use the output weight of the ELM to predict the target output *T* as follows:

(24) $$\begin{array}{c} H\hat{\beta }=T. \end{array}$$

When the activation function is *g*(*x*) and ELM chooses to add nodes, the hidden layer output matrix of ELM is

(25) $$\begin{array}{c} H\left(w_{1},\ldots ,w_{\tilde{N}},b_{1},\ldots ,b_{\tilde{N}},x_{1},\ldots ,x_{N}\right)={\left[\begin{array}{ccc} g\left(w_{1}\cdot x_{1}+b_{1}\right) & \cdots & g\left(w_{u}\cdot x_{1}+b_{\tilde{N}}\right)\\ \vdots & \cdots & \vdots \\ g\left(w_{1}\cdot x_{N}+b_{1}\right) & \cdots & g\left(w_{u}\cdot x_{N}+b_{\tilde{N}}\right) \end{array}\right]}_{N\times \tilde{N}}. \end{array}$$

When the activation function is *g*(*x*) and ELM selects the RBF kernel function, the hidden layer output matrix of ELM is

(26) $$\begin{array}{c} H\left(w_{1},\ldots ,w_{\tilde{N}},b_{1},\ldots ,b_{\tilde{N}},x_{1},\ldots ,x_{N}\right)={\left[\begin{array}{ccc} \phi \left(b_{1}\left\|w_{1}-w_{1}\right\|\right) & \cdots & \phi \left(b_{\tilde{N}}\left\|w_{1}-w_{\tilde{N}}\right\|\right)\\ \vdots & \cdots & \vdots \\ \phi \left(b_{1}\left\|w_{N}-w_{1}\right\|\right) & \cdots & \phi \left(b_{\tilde{N}}\left\|w_{N}-w_{\tilde{N}}\right\|\right) \end{array}\right]}_{N\times \tilde{N}}. \end{array}$$

The output weight of ELM is

(27) $$\begin{array}{c} \hat{\beta }=H^{+}T,\\ ={\left(H^{T}H\right)}^{-1}H^{T}T. \end{array}$$

Compared with the traditional SLFN, the ELM parameters are randomly generated, and there is no need to manually set the initial value, which reduces manual interference and reduces the time for iterative adjustment of parameters. Practical results prove that ELM has better generalization ability than gradient descent SFLN (such as BP algorithm). In terms of fast learning, especially, the learning time of certain application problems can be completed in a few seconds or even shorter. Additionally, ELM solves the traditional SLFN learning stop conditions, learning steps, and local minimization problems.

## 4. Model Building

**Figure 2:**
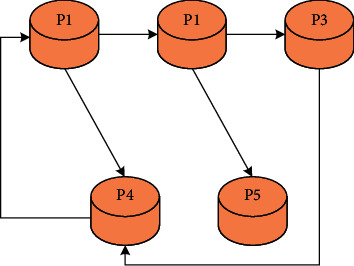
Directed graph *G*.

To describe the algorithm in this paper more vividly, the English word order can be compared to a directed graph set, which is composed of a series of web pages (analogous to the nodes of a directed graph) and hyperlinks (analogous to the arcs of a directed graph). It is particularly noted that the arc has a direction, and its direction represents the incoming chain or the outgoing chain. The directed graph *G* shown in Figure 2 represents a simple micronetwork, where P1, P2, P3, P4, and P5 represent English words, respectively.

**Figure 3:**
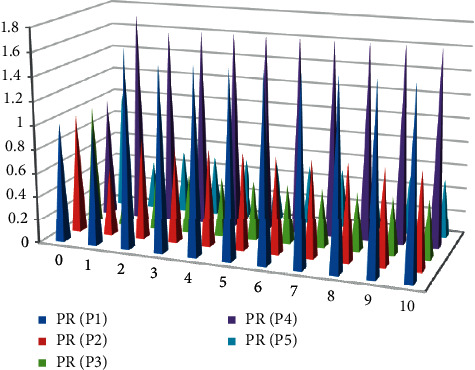
Statistical diagram of the iterative process of PR value.

This article uses the algorithm proposed in this article to calculate the PR value of the vocabulary, which is an iterative process in mathematics. The efficiency of the iterative algorithm largely depends on the preset number of iterations, so the number of iterations should be set reasonably according to the web page structure. The algorithm is applied to the network shown in Figure 2. The PR value of each word is initialized to 1, and the PR value of each word is calculated after iteration, as shown in Table 1 and Figure 3.

Through iterative operation, the PR value of the final vocabulary approaches a fixed value. Finally, we found that the PR value of vocabulary P4 is the highest, while the PR values of vocabulary P3 and vocabulary P5 are always the same during the iteration process, because the in-chain and out-chain of these two words are the same. Table 1 shows that the algorithm has converged when the number of iterations is 9, and redundant iteration steps will only reduce the efficiency of the algorithm. Therefore, it is very important to set the number of iterations of the PageRank algorithm reasonably.

**Figure 4:**
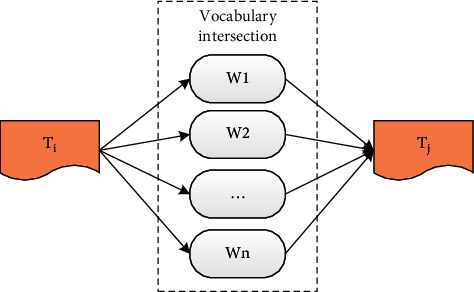
Link relationship between texts.

The text is a static structure, and it has no links. Therefore, the first problem is to find a certain connection between the texts, to explain the existence of the link relationship between the texts, so as to measure its importance. The same vocabulary between texts can be regarded as a kind of connection, and the vocabulary intersection between texts can be further regarded as a static link relationship, as shown in Figure 4.

Among them, *T*_*i*_ and *T*_*j*_ represent two texts, and *W*_1_, *W*_2_,…, *W*_*n*_ represents the vocabulary intersection between the two texts.

**Figure 5:**
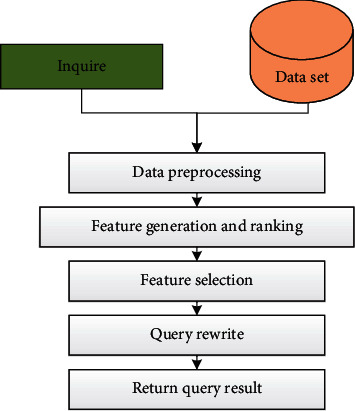
Implementation steps of query expansion.

Query expansion is two powerful core research techniques in the field of information retrieval. Its purpose is to solve the problem of short query and term mismatch and assist users to better use search engine technology to obtain the relevant information they need more easily and effectively. Query expansion is one of the indispensable processes in the field of short text retrieval, and the quality of its algorithm directly affects retrieval performance. The search result of the original query entered is the data source that generates the new expansion word. The steps of query expansion are briefly explained in Figure 5.

To improve retrieval efficiency, we need to rewrite the query and add the obtained new query words to the constructed query. The second retrieval of the traditional pseudorelevance feedback related model query uses the same algorithm as the first retrieval and does not distinguish between the newly added extended feature words and the original query words. This has not greatly improved the query accuracy. This paper proposes an improved sorting algorithm to improve the sorting calculation method in terms of content relevance and real-time performance. When calculating the score of the document, the extended word weight factor and time factor are added to improve the retrieval efficiency. The specific algorithm idea is as follows. After the extended feature words are selected in the first retrieval result, each feature word has a corresponding weight, and the weight information is added to the calculation of the second retrieval. At the same time, due to the strong real-time nature of Weibo information, it is necessary to improve the ranking of documents closer to the query time. In addition, the difference between the query time and the creation time of the document is selected as another feature, and the correlation between the query term and the document is calculated based on the previously mentioned features, and the order is sorted according to the obtained weight.

## 5. Model Performance Test Analysis

By examining the indexes of the NTF-IDF-TR algorithm and the NTF-IDF algorithm, it is verified that the algorithm proposed in this paper has a certain influence on the ranking of the English word order retrieval results. In this experiment, we use Lucene's own word segmentation tool to segment the text dataset and use the information gain method to extract text features, and the text feature dimension is set to 50. In the display of search results, a threshold can be set in the experiment, and the text will be returned only when the similarity between the query word and the text exceeds this threshold. The algorithm in this paper is named SO.

**Figure 6:**
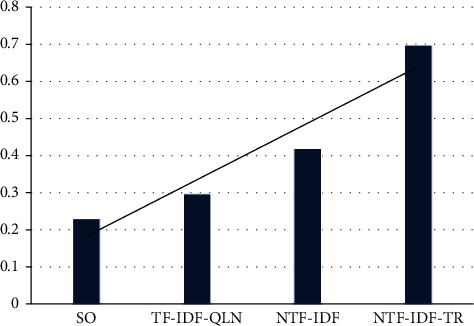
Statistics diagram of the operating efficiency of different algorithms.

This article examines the operating efficiency of different comparison algorithms. For the same experimental dataset, the same query items are input to examine the operational efficiency of each comparison algorithm. In the experiment, the threshold can also be modified to return a different number of relevant search result entries. The experimental results are shown in Table 2 and Figure 6.

It can be seen from Table 2 and Figure 6 that SO-NTF-IDF-TR is the most inefficient in terms of operational efficiency. The reason is that the SO-NTF-IDF-TR algorithm not only needs to preprocess the text, feature extraction, term weight calculation, and other common text processing tasks, but it also needs to construct a text correlation matrix based on the vocabulary intersection between texts and perform iterative operations on it to calculate the importance of each text. When the text is longer and contains more vocabulary, the amount of calculation is relatively large, and it takes more time. The difference between TF-IDF, TF-IDF-QLN, and NTF-IDF lies in the number of factors considered when calculating the weight of terms. The more factors considered, the longer the processing time. In addition, when the amount of text contained in the text collection is large, the processing time will increase accordingly.

**Figure 7:**
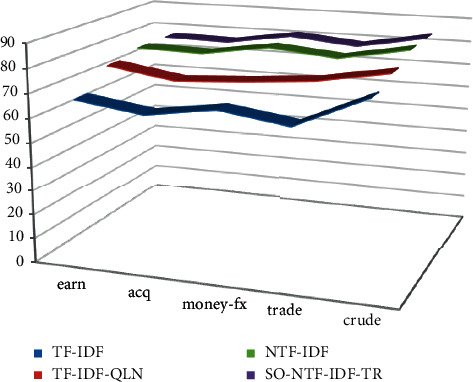
Accuracy performance.

**Figure 8:**
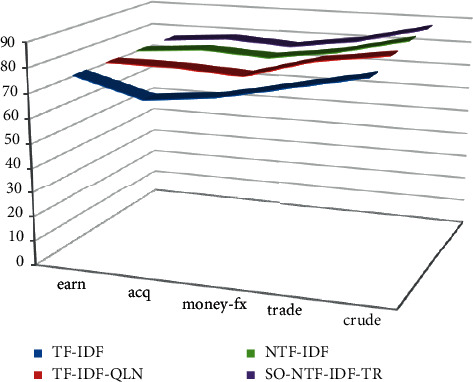
Recall performance.

**Figure 9:**
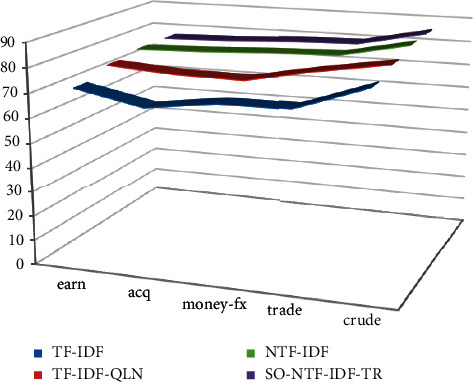
*F* value performance.

On the premise that the experimental data and related parameters are the same, enter the corresponding query items in the five categories to test each comparison algorithm. Only the texts whose similarity is greater than the set threshold are returned. The corresponding accuracy, recall, and *F* value of text retrieval in different categories are shown in Table 3. The corresponding statistical graph is shown in Figures 7–9.

The comparative experiment results show that the accuracy, recall and *F*-value of the SO-NTF-IDF-TR algorithm and the NTF-IDF algorithm are the same. Although the algorithm of this paper is integrated into SO-NTF-IDF-TR, it has nothing to do with user queries and can only change the order of the search result list. Therefore, it has no effect on the returned results of the query. These two algorithms are better than the other two algorithms in the comparison algorithm in terms of retrieval effect. The reason is that the NTF-IDF algorithm considers the length of the query keyword in the calculation of the term weight. Therefore, the weight can be smoothly assigned according to the length of the query key. At the same time, the SO-NTF-IDF-TR algorithm incorporates the algorithm proposed in this article in the display of the search result list, *t*, and considers the static relationship between the text collections, which can prevent some meaningless texts from appearing in the sorting results, so as to ensure that the improved algorithm in this paper can achieve a better recall rate under the same accuracy rate. Generally, this article has a certain effect on the improvement of the TF-IDF weighting method. The overall performance of the algorithm is better, the precision is higher, and it focuses on returning the most accurate search results to users.

When using the SO-NTF-IDF-TR method, its accuracy is improved. The reason is that, for the SO-NTF-IDF-TR algorithm, because it integrates an algorithm that considers the importance of text, the SO-NTF-IDF-TR algorithm often returns text that is closely related to the user's query in the first few items of the search list. However, as the value *N* gradually increases, the accuracy of the SO-NTF-IDF-TR algorithm gradually decreases. This is because as the number of search lists increases, there will be many intrusive texts that are irrelevant to the user's query or have low similarity in the returned search results. It only contains query keywords, which is not in line with the original intention of the user's query. Naturally, the accuracy of the returned text is not high. Therefore, when the value of *N* increases, it will affect the accuracy of the SO-NTF-IDF-TR algorithm.

## 6. Conclusion

This paper sorts the word order and vocabulary of English search and realizes the sorting purpose by constructing an artificial intelligence model. Moreover, this article proposes the SSOSELMR algorithm based on the ELM algorithm. First, for semilabeled data, this paper proposes a semisupervised ELM regression model. Then, for the semilabeled NIP spectral data of the online sequence, this paper further improves the semisupervised ELM regression model and proposes a semisupervised online sequence ELM regression model. The experiment proves that SSOSELMR semisupervised online learning effectively improves the learning ability of traditional NIR-supervised batch mode. Moreover, it introduces the text length factor into the vector space model to improve the lexical item weight measurement method and introduces the text importance measurement to examine the importance of each text in the text collection and reorder the search results. Finally, this paper compares and analyzes the weight calculation method and sorting algorithm proposed in this paper and the improved algorithm proposed by other literature through experiments. The experimental results show that the algorithm proposed in this paper effectively improves the accuracy of text retrieval and, to a certain extent, improves the ranking of the retrieval result list.

## Figures and Tables

**Table 1:** Iterative process of PR value.

	PR (P1)	PR (P2)	PR (P3)	PR (P4)	PR (P5)
0	1	1	1	1	1
1	1	0.575	0.3944	1.7602	0.3944
2	1.6627	0.8581	0.5162	1.6355	0.5162
3	1.5417	0.8067	0.4944	1.6657	0.4944
4	1.5673	0.8176	0.4989	1.6620	0.4989
5	1.5642	0.8163	0.4984	1.6641	0.4984
6	1.5659	0.8170	0.4987	1.6646	0.4987
7	1.5664	0.8172	0.4988	1.6652	0.4988
8	1.5669	0.8174	0.4989	1.6655	0.4989
9	1.5671	0.8176	0.4989	1.6657	0.4989
10	1.5671	0.8176	0.4989	1.6657	0.4989

**Table 2:** Operating efficiency of different algorithms.

Comparison algorithm	Time/s
TF-IDF	0.2303
TF-IDF-QLN	0.29694
NTF-IDF	0.41944
SO-NTF-IDF-TR	0.69776

**Table 3:** Comparison of accuracy, recall and *F* value of different algorithms.

Text set category		Earn	acq	Money-fx	Trade	Crude
TF-IDF	Number of related texts	82.5	34.1	29.7	16.5	TF-IDF
	Returns the number of texts	135.3	59.4	48.4	28.6	
	Accuracy (%)	66.99	63.14	67.54	63.47	
	Recall rate (%)	76.56	68.97	71.94	77.66	
	*F* value (%)	71.5	65.89	69.63	69.85	

TF-IDF-QLN	Number of related texts	92.4	39.6	31.9	16.5	TF-IDF-QLN
	Returns the number of texts	135.3	61.6	48.4	24.2	
	Accuracy (%)	75.13	70.73	72.49	74.91	
	Recall rate (%)	76.45	76.23	74.69	82.39	
	*F* value (%)	75.24	73.37	73.04	78.43	

NTF-IDF	Number of related texts	85.8	40.7	34.1	16.5	NTF-IDF
	Returns the number of texts	122.1	58.3	46.2	23.1	
	Accuracy (%)	77.33	76.78	81.18	78.54	
	Recall rate (%)	76.45	78.65	77	80.96	
	*F* value (%)	76.89	77.66	78.98	79.75	

SO-NTF-IDF-TR	Number of related texts	85.8	40.7	34.1	16.5	SO-NTF-IDF-TR
	Returns the number of texts	122.1	58.3	46.2	23.1	
	Accuracy (%)	77.33	76.78	81.18	78.54	
	Recall rate (%)	76.45	78.65	77	80.96	
	*F* value (%)	76.89	77.66	78.98	79.75	

## Data Availability

The data used to support the findings of this study are available from the corresponding author upon request.
